# The role of GammaTile in the treatment of brain tumors: a technical and clinical overview

**DOI:** 10.1007/s11060-023-04523-z

**Published:** 2024-01-23

**Authors:** Michael A. Garcia, Adam Turner, David G. Brachman

**Affiliations:** GT Medical Technologies, Inc., Tempe, AZ, USA

**Keywords:** Brachytherapy, Brain tumors, Glioblastoma, Brain metastases, Meningioma

## Abstract

Malignant and benign brain tumors with a propensity to recur continue to be a clinical challenge despite decades-long efforts to develop systemic and more advanced local therapies. GammaTile (GT Medical Technologies Inc., Tempe AZ) has emerged as a novel brain brachytherapy device placed during surgery, which starts adjuvant radiotherapy immediately after resection. GammaTile received FDA clearance in 2018 for any recurrent brain tumor and expanded clearance in 2020 to include upfront use in any malignant brain tumor. More than 1,000 patients have been treated with GammaTile to date, and several publications have described technical aspects of the device, workflow, and clinical outcome data. Herein, we review the technical aspects of this brachytherapy treatment, including practical physics principles, discuss the available literature with an emphasis on clinical outcome data in the setting of brain metastases, glioblastoma, and meningioma, and provide an overview of the open and pending clinical trials that are further defining the efficacy and safety of GammaTile.

## Introduction

 Brain tumors that tend to recur, such as high-grade glioma, brain metastases, and some meningiomas, inflict a great degree of morbidity and mortality. Adjuvant radiation therapy after resection of these tumors is standard of care, aimed at providing local control (LC). Despite technological advances made in external beam radiation therapy (EBRT), such as stereotactic radiotherapy (SRT), intensity modulated radiation therapy (IMRT), and protons, LC remains a challenge in the postoperative setting for brain metastases [1–3], glioblastoma (GBM) [4], and recurrent meningioma [5].

Suboptimal outcomes may continue for several reasons. The time needed for wound healing between resection and EBRT initiation allows opportunity for proliferation of residual tumor cells, which can result in rapid early progression (REP) as seen in GBM [6], and rapid recurrence (RR) of brain metastasis before initiation of adjuvant EBRT [7, 8]. Precisely defining the target volume for a postoperative tumor cavity can be challenging when using EBRT, particularly for brain metastases [9–11]. Additionally, providing LC after recurrence of a previously irradiated tumor is especially challenging since brain immediately adjacent to the local recurrence would typically have already received EBRT to near tolerance doses [12–14], making it difficult to provide efficacious doses of radiation with repeat EBRT for tumors that have shown themselves to be resistant to EBRT.

Implanting internal radioactive sources within the tumor cavity immediately after resection, a radiation modality referred to as brachytherapy (BT), may address multiple intrinsic challenges of EBRT and thus expand available options for effective postoperative adjuvant therapy [15–17]. Dose from low-energy radioactive sources implanted within the surgical cavity is characterized by a steep fall off in tissue surrounding the target volume, which results in dosimetric sparing of scalp or distal normal brain parenchyma compared to EBRT [15, 16, 18]. Among brain BT techniques, GammaTile® (GT Medical Technologies Inc., Tempe AZ) was specifically developed to improve the dosimetric, technical, and workflow aspects of existing techniques [19, 20] and is the only commercially available collagen tile BT.

Below, we review the technical aspects of GammaTile therapy and then discuss published clinical outcome data for brain metastases, GBM, and meningioma. We discuss how GammaTile use may help circumvent two management barriers that can affect outcomes: (1) time delays to EBRT (and the phenomenon REP/RR) and (2) the difficulty of treating recurrent, previously irradiated tumors. We also discuss the four ongoing and one upcoming clinical trials (Table 1) evaluating the safety and efficacy of GammaTile across various clinical scenarios.

**Table 1:** Ongoing and upcoming clinical trials involving GammaTile therapy

	STaRT registry	ROADS	Memorial Sloan kettering cancer center phase II	GESTALT	PATHWAYS
NCT #	NCT04427384	NCT04365374	NCT04690348	NCT05342883	NCT05900908
Tumor types	Brain metastases, GBM, meningioma, and all other brain tumor types	Newly diagnosed brain metastases requiring resection (2–5 cm).	Recurrent, previously irradiated metastases planned for resection	Newly diagnosed GBM	Recurrent GBM (1st recurrence)
Design	Basket, observational	RCT Surgery + GT vs. Surgery + SRS.	RCT Surgery + GT vs. surgery alone	Single arm feasibility trial. Combining GT with IMRT + TMZ	RCT (1:2 randomization) Surgery vs. surgery + GT. Both arms get Lomustine or Bevacizumab.
Planned enrollment	600	180	76	61	267
Outcomes	LC, OS, Functional Status, AEs, QOL	LC, OS, AEs KPS, Neurocognitive Status, and QOL	LC, OS, AEs, and Neurocognitive Status	Starting IMRT + TMZ ≤ 35 days post-surgery, consent and attrition rate, AEs, OS, LC	Overall Survival, LC, Neurocognitive Status, QOL, AEs
Follow up	5 years	2 years	2 years	Up to 3 years	Up to 24 months
Opened	2020	2021	2020	2022	Anticipated late 2023

Legend: *RCT* randomized control trial, *GT* GammaTile, *SRS* stereotactic radiosurgery, *IMRT* intensity modulated radiation therapy, *TMZ* temozolomide, *LC* local control, *OS* overall survival, *AE* adverse effects, *QOL* quality of life

## Technical factors

**Fig. 1:**
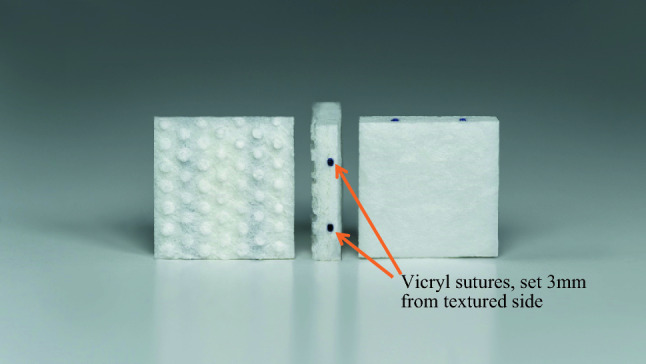
GammaTile collagen square. The GammaTile has a textured surface and smooth surface. The textured surface faces the operative bed/brain parenchyma at time of implant as this is the surface that has a 3 mm offset from the Cs-131 seed

**Fig. 2:**
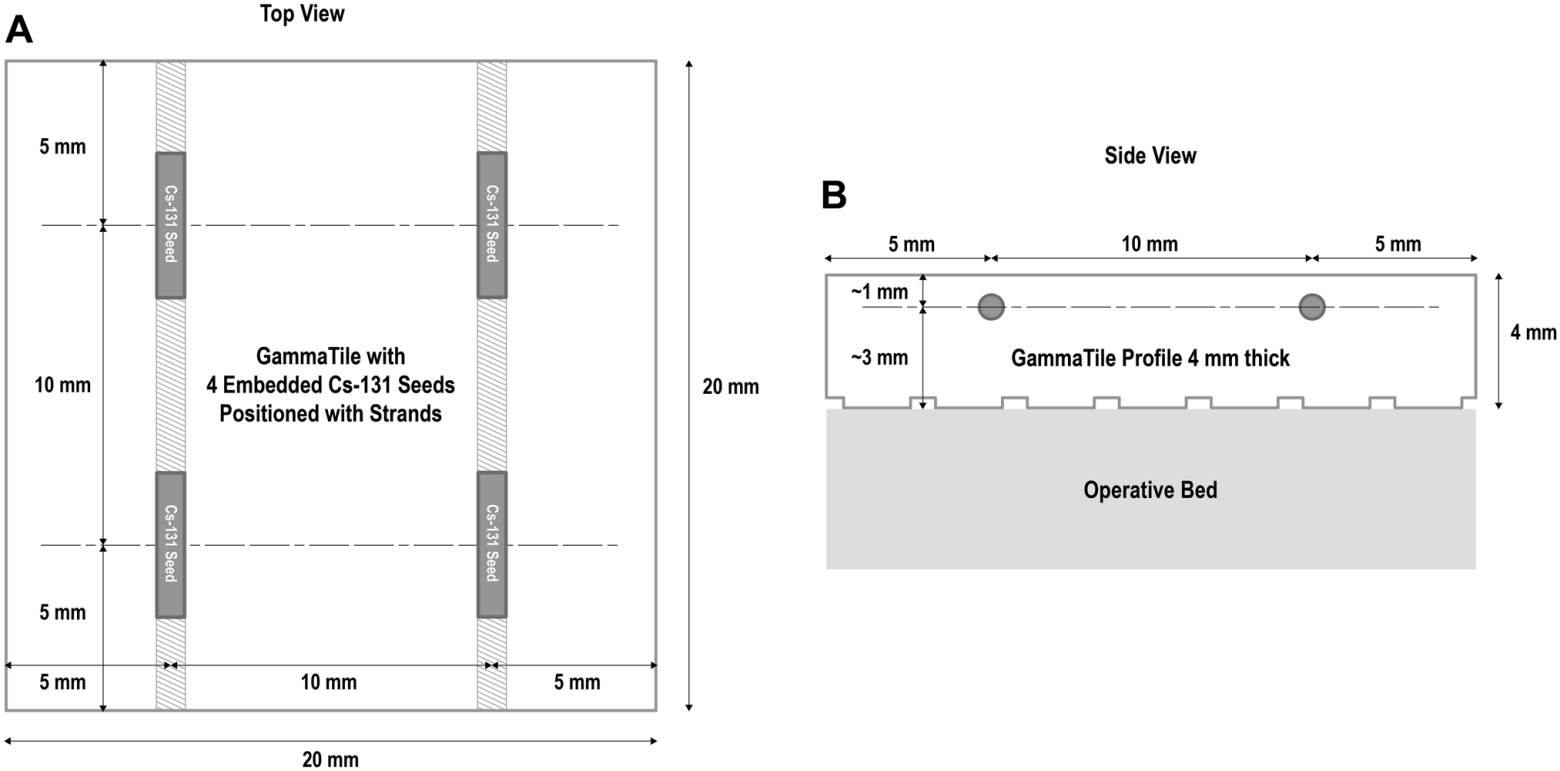
Seed spacing. Schematic of a single GammaTile. Each GammaTile is a 20 × 20 × 4 mm collagen tile and contains 4 Cs-131seeds. The center of each Cs-131 seed is spaced 10 mm apart from one another (**A**). The seeds are spaced 3 mmfrom the surface of the tile that faces the operative bed/brain tissue (**B**).

 GammaTiles are bioresorbable, conformable, 20 × 20 × 4 mm collagen squares containing four radioactive titanium-encapsulated cesium-131 (Cs-131) seeds per tile (Fig. 1). Upon completion of resection, the neurosurgeon lines the tumor cavity with sufficient tiles to cover the surfaces at risk for recurrence. This permanently implanted device functions as both a seed carrier and three-dimensional spacer, offsetting seeds 3 mm from the tumor cavity surface and 10 mm from each other (Fig. 2). The 10 mm fixed inter-seed spacing aims to prevent dosimetric “hot spots” and “cold spots” that could arise from irregular spacing of seeds and can occur with more traditional brain BT methods, such as stranded seeds [19]. The 3 mm offset between seeds and the tile surface prevents the very high doses of radiation that would occur if seeds were placed directly on brain.

**Fig. 3:**
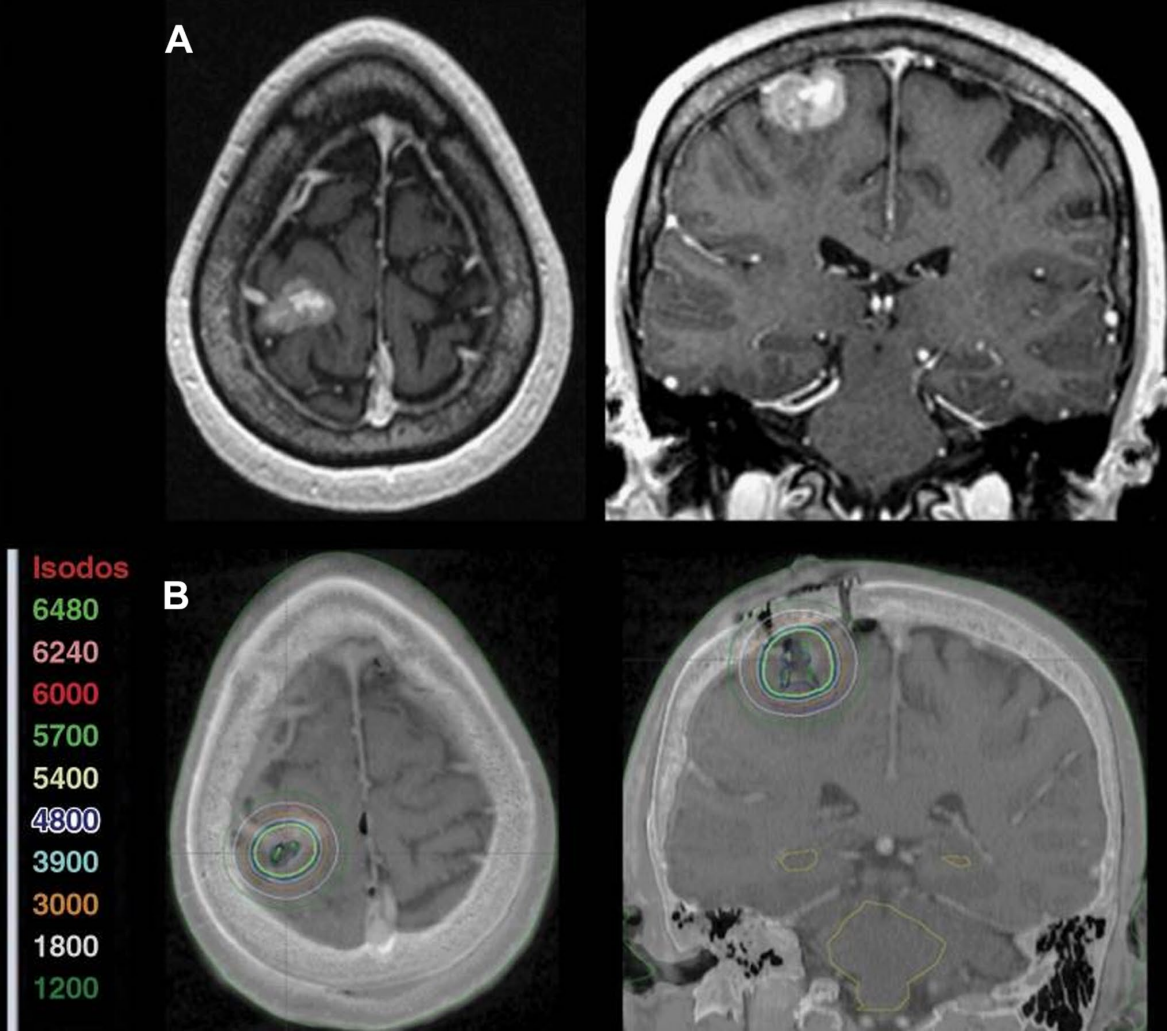
GammaTile dosimetry. **A** Preoperative T1 post contrast MRI showing a single brain metastasis. **B** Postoperative MRI fused with postoperative CT scan demonstrating GammaTile dosimetry (doses in centigray) [8]

The radioactive Cs-131 seeds used in GammaTiles are manufactured to have a fixed source strength of 3.5 U (cGy cm^2^ h^−1^) on day of implant. The GammaTile design and source strength results in a physical dose of approximately 60 Gy to a depth of 5 mm in brain (Fig. 3); however, cavity shape, cavity size, and number of tiles can affect specific implant dose distributions. Compared to more traditional brain BT using seeds with a range of source strengths and heterogeneity of prescription doses [21, 22], standardization of source strength at implant reduces dosimetric variability across patients and may simplify studying outcomes.

Cs-131 has a half-life of 9.7 days, compared to 59.4 days for iodine-125, which has also been used in brain BT. The relatively short half-life of Cs-131 ensures the planned dose is given in a shorter period at a higher initial dose rate, which may better address highly proliferative malignant brain tumors [23]. The short half-life also helps ensure the planned dose is delivered before collagen tile reabsorption and seed displacement [24–27].

## Preoperative, intraoperative, and postoperative workflow

GammaTile therapy requires a multidisciplinary care team approach [28]. Neurosurgery, neuro-oncology, and radiation oncology teams collaborate on patient selection and accurate case planning. Based on a preoperative MRI, the surface area of the expected postoperative tumor cavity deemed at risk for recurrence is estimated, considering portions of the cavity that may not need tiles (e.g., surgical tract) and potential intraoperative cavity contraction. After tumor resection, the tiles are placed on the tumor bed with the 3 mm offset from the seed facing brain parenchyma to achieve the desired seed-to-brain offset.

The collagen component of the GammaTile is the same material neurosurgeons have used to reconstruct dura for decades, and the ease of usability is demonstrated with an average implantation time of 2–5 min [24]. The fast implant time helps minimize radiation exposure to perioperative staff and limits the time patients spend under anesthesia. In a study of 22 patients treated with GammaTile (including two patients who had multiple GammaTile treatments for separate tumors), measured and modeled radiation exposure for healthcare workers and caregivers was below regulatory limits for medical personnel and the public [29].

**Fig. 4:**
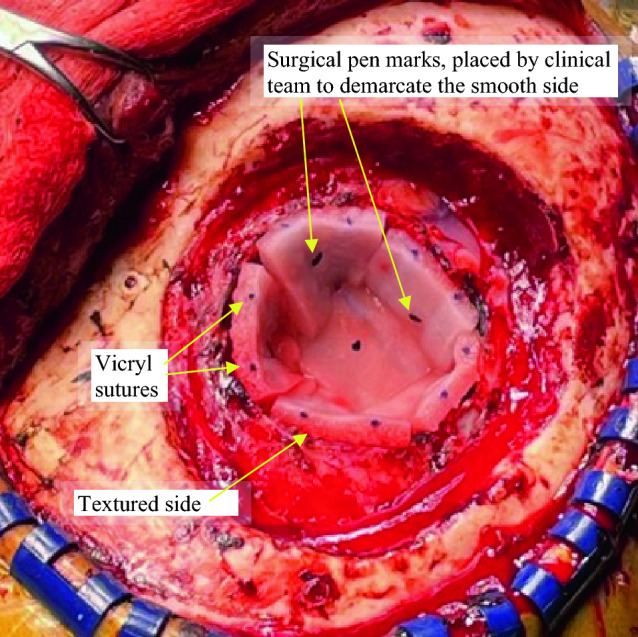
Surgery cavity lined with GammaTiles (5 along the periphery, one at the base). The smooth surface of the tile is marked with a surgical pen to ensure the textured surface faces brain at time of implant

Following surgical closure, a postoperative radiation survey measurement is performed to quantify the maximum radiation exposure one meter from the implant site to verify the patient meets regulatory limits for patient release. Using U.S. Nuclear Regulatory Commission recommendations, patients may be released based on 1-meter measurements of ≤ 6 mrem/hr [30]. In a single institution study, the reported mean survey measurement was 1.83 mrem/hr with a range of 0.5–3.5 mrem/hr for 13 patients [31]. In addition to the standard postoperative MRI exam assessing extent of resection, a thin-cut non-contrast brain CT is obtained, typically 12–72 h postimplant. Like other forms of permanent seed BT, the CT is used to document the position and number of implanted sources as well as the isodose lines/radiation field (Fig. 4) [32]. Importantly, the outcome data (LC, toxicity) described below results from surgically targeting the oncologic at-risk tissue with GammaTiles rather than trying to meet pre-specified dosimetry parameters. To date, no specific postimplant dosimetric parameters have been correlated with probability of LC or toxicity in the case of recurrent GBM [33] or brain metastases [8]. Predictive postimplant dosimetric parameters may become apparent as trial data matures (Table 1).

## Brain metastases

Brain metastases are the most common malignant brain tumors in adults, and they pose a burden for patients with cancer both at initial diagnosis and re-occurrence. The use of SRT for intact, typically smaller, non-resected brain metastases confers LC on the order of ~ 85% [34]. Durable LC in the postoperative setting for larger brain metastases requiring resection has been more challenging, with LC at 60–79% [1–3]. Whole brain radiotherapy is another option, but comes at the cost of neurocognitive toxicity and still results in a local failure rate of ~ 14% when used in the postoperative setting [1].

Intraoperative treatment with GammaTile implantation may mitigate factors that lead to worse LC after resection. Changes in the resection cavity between SRT planning, MRI, and SRT delivery could lead to a marginal miss, especially due to the highly conformal nature of SRT [7, 9]. Also, time delay from surgery to SRT initiation (which can result from a skilled nursing stay, coordination with systemic therapies, and logistics for setting up radiation oncology consultation, CT simulation, and radiation delivery [35]) leads to worse LC, with a local failure rate of 2.3% if SRT starts before 4 weeks, 14.5% between 4 and 8 weeks, and 48.5% if started after 8 weeks postoperation [35]. Some patients are lost to follow up and never receive SRT, with one prospective trial of post-operative SRS demonstrating 22% of patients never received the planned SRT course [36]. Furthermore, a subset of aggressive brain metastases can rapidly recur after complete resection but before postoperative SRT, even if SRT is delivered before 4 weeks [8]. Compared to postoperative SRT, GammaTiles are implanted at resection, guaranteeing adjuvant radiation starts immediately.

### Recurrent brain metastases

Local recurrence of a brain metastasis previously treated with radiation is a particularly challenging clinical situation. Some management options for recurrent previously irradiated brain metastases include SRT, surgery alone, and surgery followed by postoperative SRT. The 1-year estimated LC rate after repeat SRT is 61–68% [37, 38]. The frequency of adverse radiation effect in the repeat SRT (non-surgical) setting from a large series at UCSF was 37% [39], and the frequency of symptomatic radiation necrosis (RN) is 20–24% [39, 40].

Surgical resection can be done for recurrent previously irradiated brain metastases, especially when there is mass effect, but surgery without adjuvant radiation leads to a local failure rate of 40–44% [34, 41]. Postoperative SRT after resecting a previously irradiated brain metastasis leads to LC of 70–75% with an RN rate of 13% [41, 42]. Lowering the radiation dose, which can mitigate RN, may compromise LC [43], especially for radioresistant tumors (recurring after radiation).

In the initial multi-histology prospective basket trial at Barrow Neurological Institute evaluating GammaTile, 96 patients with 108 tumor cavities received resection and GammaTile. Within this cohort, 12 patients with recurrent brain metastases were enrolled. The estimated 1-year LC of recurrent previously irradiated brain metastases treated with GammaTile was 80%. The single patient that experienced local failure had a 3.2 cm sarcoma metastasis. Two patients (16.7%) experienced radiation injury that resolved with dexamethasone [20].

GammaTile outcomes in the recurrent setting have been largely reproduced on a prospective registry at Memorial Sloan Kettering Cancer Center [44], where 20 patients received GammaTile to a total of 25 brain metastases that were previously irradiated and had recurred (median recurrence was 3 cm). The 1-year local failure incidence after resection and GammaTile was 8%, and symptomatic necrosis was 16%, which was managed with dexamethasone. This registry trial prompted investigators at Memorial Sloan Kettering Cancer Center to initiate a randomized Phase 2 trial (NCT04690348) comparing resection with GammaTile to resection without GammaTile (Table 1).

Most recently, Miami Cancer Institute reported on their experience using resection and GammaTile for recurrent previously irradiated brain metastases. They compared LC after salvage surgery plus GammaTile to LC after the first course of EBRT. The 6-, 12-, and 18-month LC rates were 66.7%, 33.3%, and 25% after the previous EBRT, and in comparison, these rates were 100%, 100%, and 100% after surgery plus GammaTile (p < 0.001). At a median follow up of 14.5 months, there was one instance (8%) of RN (Grade 2) [45].

### Newly diagnosed brain metastases

As a pilot study, The University of Minnesota evaluated GammaTile for newly diagnosed brain metastases. They identified 10 consecutive patients with rapidly growing brain metastases, which they defined as either (1) brain metastases that developed symptomatic postoperative recurrence before radiation could be given (within 4 weeks of surgery) and required another resection or (2) patients who had brain metastases enlarged by > 25% volume before surgery and were considered at risk of RR between surgery and SRT. Once identified, these patients all underwent surgery (resection or re-resection) plus GammaTile. At a median follow-up of 186 days, no patients experienced local recurrence and there were no incidences of RN [8].

The use of GammaTile for newly diagnosed brain metastasis requiring resection is being evaluated by the Phase 3 trial ROADS trial (NCT04365374) (Table 1). In this trial, patients with tumors measuring 2–5 cm in diameter are randomized between surgery plus postoperative SRT versus surgery plus GammaTile. Primary outcome is LC with secondary outcomes including overall survival (OS) and rates of leptomeningeal disease (which can occur in up to 30% of cases after SRT) [46].

## Glioblastoma

GBM is the most common malignant primary brain tumor in adults. Most re-occur within 2 cm of the resection cavity [4, 24, 47–49]. Despite great efforts over the last ~ 20 years to improve the standard of care for the treatment of newly diagnosed GBM, median OS from diagnosis continues to be poor, around 16–20 months [50].

At recurrence, there is no established standard of care, and available options include surgical resection, re-irradiation, systemic therapies and/or immunotherapies, alone or in combination [12–14, 50–52]. None of the available treatment strategies have shown significant OS via randomized trials.

### Recurrent glioblastoma

As part of the multi-histology prospective basket trial at Barrow, 28 patients with recurrent GBM received maximum safe resection with GammaTile (20 patients at first recurrence, 8 patients at second or third recurrence). Median time to local failure was 12.1 months and median OS from diagnosis was 25 months [17]. There was a 7% rate of symptomatic RN among these patients managed with dexamethasone.

Another prospective cohort of patients at the University of Minnesota was treated with maximum safe resection and GammaTile for IDH wild-type GBM recurrence. 6- and 12-month LC was 86 and 81% respectively, and median OS from diagnosis was 25 months. Although RN was suspected in 4 patients (18%), upon subsequent craniotomy, pathology demonstrated recurrence. The authors also reported a contemporaneous cohort of patients treated with surgery but without GammaTile. Comparing the two cohorts, patients who received GammaTile with resection had a longer progression-free survival (PFS) (p = 0.05) and OS (p = 0.006) [33] compared to the non-GammaTile cohort. To date, the most used indication for GammaTile has been recurrent GBM [53].

The PATHWAYS trial (NCT05900908) will evaluate GammaTile for first recurrence of GBM by randomizing patients with IDH wild-type recurrent GBM to either surgery alone or surgery plus GammaTile (Table 1). Patients on both arms will then receive systemic therapy, either bevacizumab or lomustine, (decided by the treatment team). This trial is scheduled to open in the fall of 2023 with a target accrual of 267 patients and with a primary outcome of OS.

For patients with recurrent GBM, quality of life is a top priority, and by providing radiation at time of resection, BT can spare patients the time needed for daily treatments of EBRT. Quality of life is also an outcome measure on the registry and PATHWAYS trials (Table 1).

### Newly diagnosed glioblastoma

Standard of care for newly diagnosed GBM is maximum safe resection followed by chemoradiation (for patients with good performance status, treatment is 60 Gy in 30 fractions plus temozolomide) along with tumor treating fields [50]. Recently, a multi-institutional meta-analysis studied the frequency of GBM REP (the recurrence of any amount or growth of tumor) between maximum safe resection and start of EBRT. The incidence of REP was 46%, and importantly, REP was associated with worse PFS and OS [6]. Additionally, some recent reports suggest that delays between surgery and radiation may impact GBM outcomes [54, 55]. These findings suggest that starting radiation immediately at time of maximum safe resection could reduce the incidence of REP, preclude time delay to EBRT, and potentially improve outcomes.

The GESTALT trial (NCT05342883) (Table 1) is testing the safety and feasibility of combining GammaTile with EBRT and concurrent temozolomide as a way of “bridging” the patient from the time of surgery to the start of EBRT using BT to forestall progression. This study utilizes a novel voxel by voxel optimization algorithm such that the high risk and lower risk CTVs will get the biologically equivalent doses of 60 Gy in 30 fractions and 46 Gy 23 fractions [56].

### Meningioma

Meningiomas are the most common benign brain tumors in adults, but recurrence can lead to significant neurologic morbidity and be challenging to manage [19]. Recurrent meningiomas after previous radiation are especially challenging given the large and irregular radiation fields often required for treatment. Since patients can live many years after a recurrence, late toxicity from re-irradiation is an important consideration when deciding salvage treatment strategy. RN risk is often mitigated by administering repeat radiation at a reduced, possibly less effective, dose [19, 57].

On the multi-histology Barrow trial, 20 patients with recurrent previously irradiated meningiomas (20% Grade 1, 70% Grade 2, 10% Grade 3) were treated with resection and GammaTile. PFS at 18 months after salvage surgery plus GammaTile was 89%, compared to 50% PFS at 18 months following the previously attempted therapy). The rate of symptomatic RN was 10% [19].

### Harms

The RN rates associated with BT in the re-irradiation setting are listed above by histology, but it is also important to consider other potential adverse effects. These have been tracked on the multicenter STaRT multi-histology registry (NCT04427384) initiated in June 2020, which has enrolled over 250 patients among 33 centers (Table 1). Thus far, GammaTile perioperative morbidity (such as wound breakdown, cerebral edema, readmission, infection) is comparable to craniotomy without GammaTile [44, 58, 59]. There have been over 1,000 GammaTile implants to date [53]. Adverse events (AEs) for patients treated with GammaTile are tracked on the STaRT registry and by manufacturer GT Medical Technologies via post-market surveillance. Using this database, infection rates among all GammaTile cases is ~ 3% (data on file, internal memo. GT Medical Technologies), which is at the baseline infection rate reported for craniotomy without GammaTile [60].

## Discussion & conclusion

With any new technology in oncology, the development of mature data takes significant time. Multiple clinical trials are evaluating the efficacy and safety of GammaTile. Pending the completion of these trials, and 5 years after FDA clearance, we have reviewed the accumulated published data across multiple tumor types.

GammaTile is one method of utilizing Cs-131 brachytherapy sources for brain tumors. The other contemporary method that has been reported upon is implantation of vicryl sutures containing Cs-131 seed sources, without the 3-dimensional collagen tile carrier/spacer function. This is often referred to as “stranded seeds.” Table 2 lists the institutional experiences with Cs-131 that include local control and toxicity data that utilized either GammaTile or stranded seeds.

These data suggest LC outcomes with surgery and Cs-131 use for brain metastases, GBM, and meningioma seem promising, particularly in the context of other strategies currently employed for these complicated clinical scenarios and tumor types. In addition, the rate of surgical or radiation AEs after GammaTile use is similar to that of surgery alone or other forms of radiation. Taken together, GammaTile appears to be a useful adjuvant for resected brain tumors.

**Table 2:** Institutional experiences for Cs-131 brain brachytherapy that include local control and toxicity data

Study	Study design	Cs-131 form factor	Patients	Tumor type(s)	Efficacy outcome	Toxicity
Kutuk 2023 PMID: 37,722,990	Retrospective	GammaTile	10	Brain mets*	100% LC at 6, 12, and 18 months	1 case of symptomatic radiation necrosis
Bander 2023 PMID: 37,249,824	Retrospective	Stranded seeds	119	Brain mets*§ Meningiomas*§ Gliomas*§	1 year LC 84.7% for brain mets 83.3% for meningiomas 34.1% for gliomas	8.4% radiographic necrosis 11.8% wound complication
Dharnipragada 2023 PMID: 37,324,216	Retrospective	GammaTile	10	Rapidly growing brain mets	100% LC at median follow up of 6.2 months	No radiation necrosis No surgical complications
Chen 2022 PMID: 35,061,986	Retrospective	Stranded seeds	36	Brain mets* Meningiomas* Gliomas*	1 year LC 88% for brain mets 100% for meningiomas NR for gliomas	9.5% symptomatic adverse radiation effect 16.7% surgical complications
Gessler 2021 PMID: 35,088,050	Prospective	GammaTile	22	IDH wild-type Glioblastoma*	86% LC at 6 months, 81% LC at 12 months	No radiation necrosis Two surgical complications
Imber 2022 PMID: 35,896,906	Prospective	GammaTile	20	Brain mets*	8.4% 1 year progression incidence	16% symptomatic radiation necrosis
Smith 2022 PMID: 36,322,102	Prospective	GammaTile	28	Glioblastoma*	Median time to local failure was 12.1 months	7% rate of symptomatic radiation necrosis
Nakaji 2020 PMID: 33,224,684	Prospective	GammaTile	11	Brain mets*§	83% LC at 12 months	12.5% radiation brain changes
Xia 2018 PMID: 30,280,070	Retrospective	Stranded seeds	9	Brain mets*	LC 100% at a median follow-up of 9.4 months	No radiation necrosis
Brachman 2018 PMID: 30,579,269	Prospective	GammaTile	19	Meningioma*	PFS 89% at 18 months	10% radiation necrosis 10% patients with surgical complications

Legend: *Recurrent, §Newly diagnosed, *§Recurrent and newly diagnosed, *LC* local control, *LF* local failure, *PFS* progression free survival
